# Opportunities and challenges of a dynamic consent-based application: personalized options for personal health data sharing and utilization

**DOI:** 10.1186/s12910-024-01091-3

**Published:** 2024-08-31

**Authors:** Ah Ra Lee, Dongjun Koo, Il Kon Kim, Eunjoo Lee, Sooyoung Yoo, Ho-Young Lee

**Affiliations:** 1 Office of eHealth Research and Business, Seoul National University Bundang Hospital, 172, Dolma-ro, Seongnam-si, 13605 Gyeonggi-do Republic of Korea; 2 Interdisciplinary Program in Bioengineering, Seoul National University, 1, Gwanak-ro, Seoul, 08826 Seoul Republic of Korea; 3 School of Computer Science & Engineering, College of IT Engineering, Kyungpook National University, 80, Daehak-ro, Daegu, 41566 Daegu Republic of Korea; 4 College of Nursing, Research Institute of Nursing Science, Kyungpook National University, 680, Gukchaebosang-ro, Daegu, 41944 Daegu Republic of Korea; 5 Department of Nuclear Medicine, Seoul National University Bundang Hospital, 172, Dolma-ro, Seongnam-si, 13605 Gyeonggi-do Republic of Korea

**Keywords:** Dynamic consent, Data sovereignty, Autonomy, Personalized, Personal health data

## Abstract

**Background:**

The principles of dynamic consent are based on the idea of safeguarding the autonomy of individuals by providing them with personalized options to choose from regarding the sharing and utilization of personal health data. To facilitate the widespread introduction of dynamic consent concepts in practice, individuals must perceive these procedures as useful and easy to use. This study examines the user experience of a dynamic consent-based application, in particular focusing on personalized options, and explores whether this approach may be useful in terms of ensuring the autonomy of data subjects in personal health data usage.

**Methods:**

This study investigated the user experience of MyHealthHub, a dynamic consent-based application, among adults aged 18 years or older living in South Korea. Eight tasks exploring the primary aspects of dynamic consent principles–including providing consent, monitoring consent history, and managing personalized options were provided to participants. Feedback on the experiences of testing MyHealthHub was gathered via multiple-choice and open-ended questionnaire items.

**Results:**

A total of 30 participants provided dynamic consent through the MyHealthHub application. Most participants successfully completed all the provided tasks without assistance and regarded the personalized options favourably. Concerns about the security and reliability of the digital-based consent system were raised, in contrast to positive responses elicited in other aspects, such as perceived usefulness and ease of use.

**Conclusions:**

Dynamic consent is an ethically advantageous approach for the sharing and utilization of personal health data. Personalized options have the potential to serve as pragmatic safeguards for the autonomy of individuals in the sharing and utilization of personal health data. Incorporating the principles of dynamic consent into real-world scenarios requires remaining issues, such as the need for powerful authentication mechanisms that bolster privacy and security, to be addressed. This would enhance the trustworthiness of dynamic consent-based applications while preserving their ethical advantages.

**Supplementary Information:**

The online version contains supplementary material available at 10.1186/s12910-024-01091-3.

## Background

The advances in big data necessitate a complicated balance between protecting the privacy of individuals whose data are being used and leveraging the societal benefits provided by state-of-the-art data-driven technologies [1]. Personal health data are a valuable resource that significantly impacts biomedical research and digital health ecosystems [2]. The integration of sophisticated technologies with the widespread use of personal health data has resulted in groundbreaking work within the realm of medicine and tangible applications in the health care sector [3]. However, the combination of technology and personal health data has led to concerns associated with data privacy and security, as well as ethical implications in terms of consent and potential exploitation [4, 5]. Therefore, to encourage innovation and enhance healthcare outcomes through the use of data, the perspectives of both data subjects and consumers, whose interests sometimes conflict, must be thoroughly considered.

Data sovereignty is indispensable within a data-driven economy [6]. This concept emphasizes the need for data subjects to have control over the use of their shared data. The absence of such sovereignty could hinder the advancement of the data-driven economy by decreasing the desire for data sharing and utilization [7]. To ensure the full potential of data utilization, discussions regarding sovereignty have evolved in the digital era. The protection of individual rights and the promotion of trust in data sharing environments are both mandatory in certain regulatory frameworks, such as the General Data Protection Regulation (GDPR) and the Health Insurance Portability and Accountability Act (HIPAA) [8, 9]. For instance, the fundamental tenet of the European Union data protection law is that individuals have authority over the sharing of their personal health data [10]. Provisions granting access, erasure, and transfer rights for personal data in specific circumstances in the GDPR help facilitate its fundamental aim of protecting data subjects. This current shift towards giving individuals autonomy over their data highlights the significance of data sovereignty in contemporary discussions in digital health ecosystems.

While obtaining consent from individuals before using their personal health data is generally crucial in clinical research, it may not always be feasible in every situation. Appropriate safeguards and ethical considerations should be implemented to protect individuals’ privacy in such cases. The fundamental basis of consent is respecting individual autonomy [11]. The Declaration of Helsinki and the Belmont Report aim to prevent exploitative and manipulative practices in clinical and medical research, and both highlight the importance of autonomy [12, 13]. Safeguarding autonomy involves more than just preventing manipulation; it also entails offering guidance and support for making autonomous decisions. These ideas have been incorporated into practice as informed consent, which includes providing comprehensive and precise information to empower individuals to make voluntary decisions [14, 15]. The All of Us research program in the United States provides individuals with adequate information to make well-informed decisions concerning their participation [16, 17]. This program ensures that potential participants are motivated to join based on their personal interests and the inherent value of their involvement by providing comprehensive details regarding program operations. This approach ensures that individuals make informed decisions according to their preferences. The Guidelines for Tailoring the Informed Consent Process in Clinical Studies (i-CONSENT guidelines) also emphasize the significance of implementing comprehensive and individualized consent procedures [18]. These guidelines advocate for ongoing, two-way communication, initiated at the outset of participant engagement and sustained throughout the duration of the study.

Dynamic consent, an innovative principle that emphasizes the protection of the data sovereignty of individuals, has attracted considerable interest [19]. Many academic studies have examined the potential benefits of dynamic consent, specifically regarding its ethical advantages in comparison to conventional consent methods [20–24]. Due to its functionality within digital interfaces that enable uninterrupted communication between data subjects and consumers, irrespective of temporal and spatial constraints, dynamic consent is regarded as the most appropriate approach for acquiring consent in digital health ecosystems [25]. Furthermore, dynamic consent provides a variety of personalized options for individuals to enhance their autonomy and self-determination with respect to the sharing and utilization of personal health data. Establishing resilient mechanisms through which individuals can exert authority over their personal health data while maintaining continuous communication is essential in the pursuit of genuine informed consent in digital settings. Nevertheless, further considerations of personalized options are still required [26, 27]. To assess the efficacy, usability, and ability to uphold individual autonomy of personalized options that are supported by dynamic consent principles, additional investigation is needed.

Therefore, in this study, user experiences based on dynamic consent principles are examined, specifically focusing on sovereignty over health data usage in various settings with personalized options. The evaluation was conducted using MyHealthHub, a digital consent application developed in this study based on dynamic consent principles. The primary objectives of this study are (1) to explore the viewpoints of individuals on the sharing and utilization of personal health data and (2) to assess user acceptance of MyHealthHub as a means for managing data sovereignty in a tailored manner while respecting individual autonomy. This study specifically focuses on individual patients, who are the principal subjects of personal health data. To analyze user acceptance, this study employed the Technology Acceptance Model (TAM), which has been widely used to understand user acceptance of information technology [28]. This study contributes to the exploration of processes to ensure data sovereignty with dynamic consent in the health care sector by examining user experiences associated with the MyHealthHub application, which facilitates the sharing and utilization of personal health data with personalized options in a tailored manner.

## Methods

### Study design

This study utilized a mixed-methods design, incorporating both a system usability test and questionnaires. MyHealthHub, a digital consent application designed in adherence with dynamic consent principles, was specifically developed for this study to facilitate the usability test. The study participants were provided with access to the MyHealthHub application, which facilitates experiences in a personalized data sharing process using virtual health data. The questionnaire included one open-ended item to elicit a wide range of perspectives from the participants, as well as multiple-choice items. The entire procedure was completed consecutively in a single session and adhered to the required ethical protocols under the necessary ethical clearance of informed consent from the Institutional Review Board of Kyungpook National University (KNU) (KNU IRB No. KNU-2021-0158).

### Participant recruitment

Participants for this study were recruited through email invitations. Potential participants were defined as individuals who have interests or experiences in digital health services and were likely to utilize digital consent applications to generate personal health data during their daily lives and to share and utilize their data. Participants were eligible if they were 18 years of age or older, resided in South Korea, had internet access on personal devices, and were proficient in using websites for various activities, such as online shopping and internet banking. The potential participants were provided with basic information materials regarding the study through email. Email addresses of potential participants were obtained through the Smart Health Standards Forum, an organization supporting smart health standards and industry development. They were encouraged to voluntarily reach out to our research team to arrange an appointment if they were interested in participating. All participants provided informed written consent and received a gift voucher as compensation upon completion.

### System usability test

**Fig. 1:**
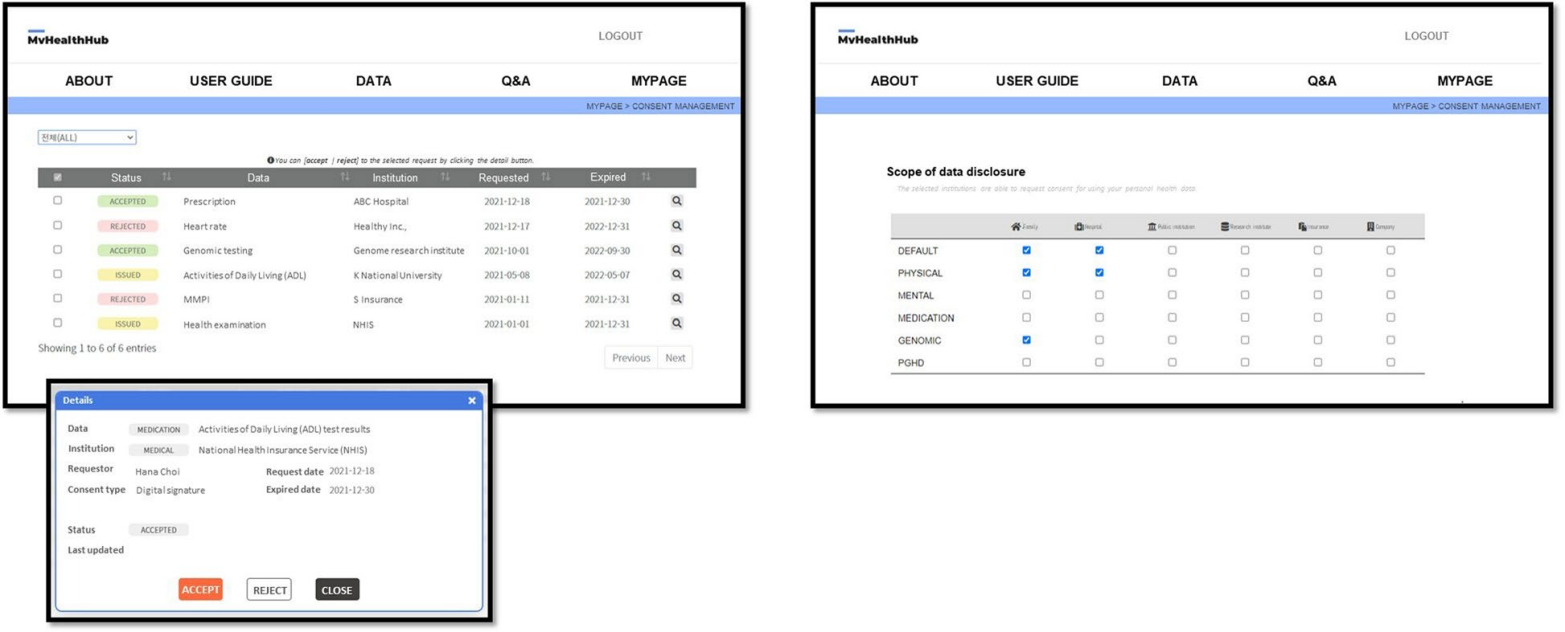
Screenshots of the English version of the MyHealthHub application

MyHealthHub is a digital consent application designed based on dynamic consent principles. This application offers participants an all-encompassing experience of personalized data sharing and consent management. The prototype version of the application was available in the Korean language. The MyHealthHub application included functionalities for managing consent, monitoring data sharing history, and configuring personalized options regarding data usage (Fig. 1). Personalized options include specifying the scope of shared data according to the specific institutions and health data involved, conditions for automatic consent, designated representatives if necessary, and preferred communication methods or periods for receiving relevant updates on their data usage. These options were flexible and could be adjusted according to each individual’s preferences.

The participants were provided with individual accounts and were instructed to access and log in to the MyHealthHub application. For the system usability test, the provided accounts were populated with temporary log-in credentials and comprised a variety of fictitious dummy personal health data. So that the participants could experience and understand the foundational attributes of dynamic consent, they were required to complete eight tasks via the MyHealthHub application. These tasks were determined through a combination of literature findings and insights from a scoping review on dynamic consent, as detailed in our previous study [29]. The tasks included providing consent, monitoring data usage history, and configuring personalized options. This approach ensures that the tasks reflected prior knowledge on dynamic consent, allowing for a comprehensive evaluation of the dynamic consent process. The participants were not specifically instructed to meticulously scrutinize each item of content available in the application during the execution of the designated tasks with the purpose of assessing real user interactions with MyHealthHub. The participants were given the autonomy to select the scope and variety of institutions that they wanted to share their data with, based on their individual preferences. After completing the tasks, participants evaluated their experience with a questionnaire designed to capture their feedback on the usability and functionality of the application.

### Questionnaire

After the usability test, participants were prompted to complete a questionnaire. The questionnaire consisted of 30 items, which comprised a combination of multiple-choice and open-ended inquiries (Table 1). The multiple-choice questions were designed to investigate perceptions of the sharing of personal health data and to assess user acceptance of the MyHealthHub application. The questionnaire items were formulated by integrating findings from the literature and primary concepts derived from the TAM, specifically focusing on perceived ease of use, perceived usefulness, and intention to use. While advancements in the model, such as the Unified Theory of Acceptance and Use of Technology, are acknowledged, this study employed the original TAM for its simplicity and well-established use in similar contexts, which aligns well with the specific focus of this study [30]. Finally, participants were provided with an open-ended questionnaire to further explore their experiences and perspectives concerning the MyHealthHub application. The participants were encouraged to provide feedback on a multitude of application-related topics, such as interface design, content, usability, potential improvements, information quality, authentication and authorization procedures, and any other pertinent observations derived from the usability test. The full version of the questionnaire was originally written in Korean, and the English-translated version is available in Additional file 1.

**Table 1:** Overview of questionnaire items

Category	Type	Number of items
1. Demographics	Multiple-choice	5
2. Perceptions on personal health data sharing	Multiple-choice	3
3. User acceptance of MyHealthHub application	Likert 7-scale	
a. Individual characteristics		
*– Health literacy*		3
*– Health-related interest*		3
b. System characteristics		
*– System usability*		3
*– System reliability*		3
c. Willingness to use		
*– Perceived usefulness*		3
*– Perceived ease of use*		3
*– Intention use*		3
4. Overall experiences		1

### Data analysis

To validate the responses gathered from the multiple-choice questionnaire items, statistical methods were utilized. The internal, concentration, and discriminant validity of each category were validated in this process [31–33]. Subsequently, descriptive statistics were employed to analyze quantitative data derived from the responses to multiple-choice questions. The qualitative data acquired through the open-ended item were analyzed thematically [34]. Two of the authors first carried out the review, coding, and categorization of the gathered data to construct the initial themes. Then, all the authors reviewed and discussed the themes to enhance their coherence and reasonability. Discrepancies identified among the authors were discussed and resolved. The data analysis procedure was performed using SmartPLS 3.0 and Excel.

## Results

### Perceptions of the sharing and utilization of personal health data

A total of thirty participants accessed the MyHealthHub application for an average of thirty minutes to provide dynamic consent. The participants successfully accomplished the eight assigned tasks without requiring additional assistance or support. The demographic characteristics of the participants are available in Additional file 2.

Table 2 presents the participants’ perspectives on the sharing of personal health data. Twenty-four out of thirty participants agreed that the exchange of personal data is crucial to the advancement of the health care industry. Regarding the timing of requesting consent, twelve participants responded that consent is needed for each time data is to be shared, whereas three participants preferred providing consent only once, such as during the initial registration process for a specific service such as the MyHealthHub application. The remaining half of the participants indicated a preference for different frequencies of consent requests, contingent upon the purposes and subjects of data usage.

**Table 2:** Perspectives on personal health data utilization

Questionnaire item	Responses (n=30)
Extent to agree with the importance of personal health data utilization for industry growth	
Strongly agree	6
Agree	7
Somewhat agree	11
Neutral	5
Somewhat disagree	1
Disagree	0
Strongly disagree	0
Frequency of required consent for data sharing and utilization	
Once (*e.g., at the initial time of registering a specific service*)	3
Partially (*e.g., various according to data usage*)	12
For each time when sharing data	15

Table 3 presents the participants’ preferences concerning the sharing and utilization of their personal health data. Participants’ degrees of willingness to share their data varied by the type of institution or data. The average number of participants who expressed willingness to share basic health checkup data was 12.17, the highest result of any data type. In contrast, the average value for data concerning mental health was the lowest, at 9.83 individuals. Regarding the institution types, an average of 26.33 individuals considered medical institutions to be favour targets for data sharing. Additionally, private companies were given the lowest preferences, with an average of only 2.00 individuals.

**Table 3:** The willingness to share personal health data by institution and data type

	Medical institution	Family	Research institute	Insurance company	Public institution	Private enterprise	Average
Basic	27	18	11	10	5	2	12.17
Physical	28	19	9	8	5	2	11.83
Genomics	27	21	12	5	4	1	11.67
Medications	28	16	9	7	4	2	11.00
Lifelogs	22	15	11	5	6	4	10.50
Mental	26	18	7	5	2	1	9.83
Average	26.33	17.83	9.83	6.67	4.33	2.00	

Basic=Results of basic health examinations, such as height, weight, vision, hearing, blood pressure, smoking history, and use of alcohol; Physical=Physical health status-related information, such as chronic disease and activities of daily living test results; Genomics=Omics-related information, such as genetic diseases and family histories; Medication=Prescription histories; Lifelogs=Person-generated health data collected from mobile devices during daily life, such as walking, heart rate, physical activity, and diet; Mental=Mental health-related information, such as psychological test results and cognitive function test outcomes

### User acceptance of the MyHealthHub application

Table 4 presents the descriptive statistics of participant responses regarding the level of user acceptance of the MyHealthHub application. The responses were validated for internal consistency, convergent validity, and discriminant validity within each category (Additional file 3). Average scores of 6.10 and 5.62 out of 7.00 were obtained for self-evaluated health literacy and health-related interests, respectively. An average of 5.67 was obtained for system usability, whereas a lower average, 4.67, was obtained for system reliability. The average score for the overall intention to use was 5.26, with 5.20 for perceived usefulness and 5.46 for perceived ease of use.

**Table 4:** Overall evaluation results for user acceptance of the MyHealthHub application

Category	Item	Average	Category average
Health literacy (HL)	HL1	5.93	6.10
	HL2	6.43	
	HL3	5.93	
Health-related interest (HI)	HI1	6.10	5.62
	HI2	4.57	
	HI3	6.20	
System usability (SU)	SU1	5.67	5.67
	SU2	5.57	
	SU3	5.77	
System reliability (SR)	SR1	5.03	4.67
	SR2	4.43	
	SR3	4.53	
Perceived usefulness (PU)	PU1	5.40	5.20
	SR2	5.33	
	SR3	4.87	
Perceived ease of use (PE)	PE1	4.80	5.46
	PE2	5.63	
	PE3	5.93	
Intention to use (IU)	IU1	5.17	5.26
	IU2	5.40	
	IU3	5.20	

### Thematic analysis results

#### Overview

Following an analysis of the responses to the open-ended questionnaire item, three themes were identified: the usability of the MyHealthHub application, the usefulness of the MyHealthHub application, and apprehensions regarding digital environments (Table 5).

**Table 5:** Summary of thematic analysis results

Theme	Subthemes identified
The usability of MyHealthHub application	• Mobile environments
	• User-friendly design
	• Intuitive interface
The usefulness of MyHealthHub application	• Personalized options
	• Autonomy
	• Self-determination
	• Inducements for data sharing
Apprehensions regarding digital environments	• Data protection
	• Transparency in data management
	• Communication strategy to build trust with end-users

#### The usability of the MyHealthHub application

In addition to the quantitative results presented in Table 4, the overall evaluation results for usability were favorable, as evidenced by the fact that every participant independently completed the assigned tasks. Moreover, participants shared some opinions about enhancing the usability of the MyHealthHub application.

Some participants believed that mobile interfaces would offer greater benefits than web-based environments. Although the prototype distributed to the participants was compatible with desktop and mobile devices, it did not have responsive interface capabilities catering to different device types. Some participants expressed the opinion that the width of the tables utilized to present a record of consent requests or data usage history was excessively large on mobile devices, requiring them to scroll to cover the entire piece of information. They expressed their desire for an iteration of the MyHealthHub application that incorporates user-interface optimization tailored for mobile devices, thereby augmenting accessibility and enabling its utilization from any location without relying on desktop computers.

In addition to mobile optimization, participants commented on the intuitiveness of the interface. The majority of participants expressed satisfaction with the level of information that the MyHealthHub application provided in relation to their decision-making process regarding data usage. On the other hand, certain participants who perceived themselves to be deficient in providing information reported facing challenges in understanding content that included medical terminology. They desired further user interface enhancement through the addition of straightforward icons or descriptions to assist them in making decisions based on a comprehensive understanding of the data types to be shared and the specific purposes for which institutions would utilize the data.

#### The usefulness of the MyHealthHub application

The participants expressed contentment with the ability to tailor the level of data sharing in accordance with the type of institutions and data. In addition, the study participants highlighted the potential advantages of the MyHealthHub application in health management, monitoring chronic diseases, and insurance payment processing.

Personalized options were found to be the most appealing aspect to participants. Furthermore, this feature complies with dynamic consent principles, which safeguard the autonomy and self-determination of individuals. Some participants who initially expressed a preference for providing consent only once during the registration procedure felt that the option to automatically set conditions for providing consent was quite attractive. Certain participants opined that a more granular degree of options would be beneficial in the selection process for institution types. For example, a participant expressed a preference for choosing a specific insurance company rather than the institution type when they desired to share data with only insurance company A but not insurance company B. On the other hand, a few participants felt that the administration of personalized options was occasionally cumbersome, impeding their motivation to engage in the process of data utilization.

The majority of participants acknowledged the benefits associated with exercising control over data through the MyHealthHub application. They valued the convenience of monitoring their consent and data usage history to help manage their data utilization, in addition to the ability to tailor the extent of shared data to their preferences. Conversely, a subset of the participants conveyed a feeling of inadequate motivation to use the MyHealthHub application. They stated that they were young and currently in excellent health, resulting in a lack of need to manage personal health data, in contrast to financial management services. Some of them suggested that it would be advantageous to employ rewards or incentives as a means of motivating individuals to share their personal health data, thereby fostering their interest in health data management.

#### Apprehensions regarding digital environments

While the participants expressed contentment with the personalized options, there were some concerns regarding security. The participants highlighted the significance of establishing security protocols to prevent disastrous data breaches in digital environments, with a particular focus on health data that may include sensitive personal information.

In response to the identification procedure, the participants provided mixed responses. The participants were able to access the MyHealthHub application through the login ID and password that were assigned for the usability test in this study. Some participants conveyed a desire to enable effortless login via the single sign-on (SSO) approach in practical situations. These participants were aware of the SSO procedure, which is an authentication approach that enables individuals to access different services with a single set of login credentials [35]. They perceived the SSO as a dependable and practical approach to accessing multiple applications, owing to its widespread adoption across various services. Conversely, certain participants expressed that they might be hesitant to use the MyHealthHub application in the future out of apprehension, citing a need for increased security measures. One participant suggested that enhanced security technologies be implemented at a level comparable to the authentication process utilized in financial applications, such as two-factor authentication [36].

An additional noteworthy opinion concerned the criticality of communication in fostering relationships of trust with system end-users. The majority of participants indicated that the functionalities provided by the MyHealthHub application are advantageous for safeguarding individual autonomy and ensuring data sovereignty over their health data. However, they also emphasized the need to provide more comprehensive information regarding data management procedures so as to enhance transparency. One participant underscored the importance of secure and permanent deletion of shared data once a contractual period has expired. Another participant contended that it is critical to convey both technical and emotional aspects pertaining to the secure storage and management of data to foster trust and assurance with system users. For instance, individuals may want to know how their provided data are transmitted to the designated institution in a secure way and how the shared data are protected within the institution.

## Discussion

This study investigated the potential of a digital consent system that adheres to dynamic consent principles for safeguarding the autonomy and data sovereignty of individuals regarding their personal health data. Dynamic consent is an innovative approach to facilitating digital health ecosystems that helps balance the use of personal health data while simultaneously safeguarding individual autonomy [19]. Previous scholarly investigations have explored dynamic consent, such as its conceptual evolution, user acceptance, and technological advancements that facilitate its practical implementation [29]. Although previous research has recognized the ethical benefits of dynamic consent in comparison to conventional consent models, a need to assess user acceptance of systems based on dynamic consent for its practical use has been consistently expressed. Notably, very few publications have linked the TAM to dynamic consent, highlighting the originality of this study in understanding user acceptance within the context of personal health data management.

The results of this study provide valuable insights into participants’ preferences and perceptions regarding the sharing and utilization of personal health data through dynamic consent mechanisms. The study demonstrated a strong acceptance of the MyHealthHub application, with participants successfully completing tasks and expressing a preference for personalized consent options tailored to the type of data and institutions involved. Notably, participants showed a higher willingness to share data with medical institutions compared to private companies, and there was a clear preference for dynamic consent methods that allow for continuous and adaptable consent management. Despite the favorable reception, some participants indicated that the abundance of options could be cumbersome, suggesting the need for further refinement of user interfaces and the incorporation of more intuitive design elements. Additionally, participants highlighted concerns about security and the importance of transparent data management practices, underscoring the necessity for robust security measures and clear communication to build trust.

In particular, there has been little effort to investigate whether dynamic consent genuinely upholds individual autonomy through the sharing and utilization of personal health data. This critical question is central to the ethical considerations of digital health technologies and the protection of individual rights and privacy [37]. It is imperative to assess the efficacy of dynamic consent in preserving these principles amid the complex interplay of technology, healthcare delivery, and individual rights [38, 39]. Individuals should be able to modify and update their consent, including actions such as protocol shifts, alterations, and withdrawal [40]. Furthermore, addressing concerns about the temporal aspect and control over the pace of interaction is essential for maintaining individual autonomy.

The personalization and flexibility of consent are enhanced by dynamic consent principles, which permit individuals to modify their consent preferences as circumstances change. This study used a digital consent application, MyHealthHub that operates on dynamic consent principles. MyHealthHub enables continuous interaction with participants, promoting self-determination in accordance with their consent preferences. The study participants comprehended and accepted the dynamic consent model according to their performance on the usability test. Participants were able to make decisions regarding the sharing of data in accordance with their individual preferences, considering the information at their disposal regarding data usage, including target institution, purpose, and duration of data sharing. Additionally, the questionnaire responses revealed that the perceived usefulness and ease of use of the MyHealthHub application led to positive intentions to use it.

The findings from this study indicate the usefulness of dynamic consent by demonstrating that individuals’ preferences regarding consent are substantially affected by a range of factors, including the kind of data to be shared, the type of institution involved, and the context in which the data is shared. These findings align with the observations made in prior studies regarding individuals’ perspectives on the utilization of their health data [41–44]. One study has indicated that individuals may exhibit a preference for providing limited data to for-profit enterprises [45]. Similarly, significant disparities in consent preferences were observed based on the type of institution in this study. The participants exhibited a greater propensity to provide consent for the sharing of their data with medical institutions or research institutes than with private enterprises. The type of health data also influenced the participants’ inclination to share their data. There was a heightened reluctance to share mental health-related data with specific institutions compared to basic health check-ups and physical health-related data.

Another notable observation from this study is that the participants displayed a mixed reaction to the personalized options. The majority of participants expressed satisfaction with the ability to independently determine the scope and extent of their data sharing, allowing customization. Some participants expressed that the abundance of options available may deter individuals from engaging in their data sharing and utilization processes. They exhibited a greater preference for automatic consent, as it eliminates the need for frequent decision-making or consent provision. The expanded role of individuals in the dynamic consent approach with respect to conventional consent mechanisms may be perceived as burdensome due to the multitude of options available for selection [46]. This particular concern has been identified as a significant barrier to the widespread adoption of the dynamic consent model in prior scholarly investigations. However, it has been argued that these opinions stem from a misinterpretation of dynamic consent. The concept of autonomy, as outlined in the dynamic consent principles, pertains to the ability to adapt approaches to accommodate various circumstances. This includes allowing individuals to choose the level of involvement they wish to have in their data-sharing processes. For example, passive individuals have the option to utilize broad-informed consent as a means to adopt a more inclusive approach within the framework of the dynamic consent model.

There are several limitations to this study. The recruitment of participants was conducted via convenience sampling. Convenience sampling was carried out by distributing invitations to individuals who were easily accessible and met the study criteria, such as members or subscribers of smart health standard forum. The majority of the study participants expressed interest in utilizing digital health services and personal health data. In fact, since this innovative method, the dynamic consent mechanism, affects the entirety of society, it is crucial to solicit the general public’s opinion. However, despite the satisfactory validity of the questionnaire responses, which suggests their potential for future research, the sample size employed in this study was relatively modest. It was difficult to recruit many public individuals, as well as older individuals, in our sample due to recruitment challenges; consequently, the characteristics of this study sample may not be representative of the general population in South Korea. The presence of such a selection bias may lead to overly optimistic conclusions regarding the level of interest and engagement of participants in utilizing the application.

Additionally, this study did not evaluate uninterrupted communication, a critical component of dynamic consent. The average duration of the participants’ experience was only 30 minutes, which is insufficient for a thorough evaluation. It is imperative to evaluate whether consent is altered over an extended period and whether participants prefer to continue utilizing the system. This limitation should be recognized, as it affects the comprehension of the continuous interaction necessary for dynamic consent systems. Furthermore, the experience was constructed using fictitious data rather than the actual data of the participants, which could potentially influence their responses and engagement. These aspects should be the focus of future research in order to conduct a more comprehensive assessment of dynamic consent systems.

Given the potential for data sharing to expand globally, it is required to address the specific contents of dynamic consent items. Previous studies have defined these items using the Data Use Ontology (DUO) developed by the Global Alliance for Genomics and Health (GA4GH) [47–49]. Additionally, international standards such as the Fast Healthcare Interoperability Resources (FHIR), developed by Health Level Seven (HL7), offer structured standards for representing consent directives in healthcare, emphasizing the importance of interoperability and consistency [50]. The Basic Patient Privacy Consents (BPPC) profile by Integrating the Healthcare Enterprise (IHE) also provides a mechanism for managing patient privacy consents, further supporting the need for standardized approaches [51]. Standardizing and clearly defining the items within dynamic consent, including the types of data shared, the purposes of data use, and the entities involved, is crucial for establishing trust among users and ensuring transparency. However, this study primarily focused on the user experience and did not explore the detailed standardization and definition of dynamic consent items, which constitutes a limitation. Addressing these aspects would enhance the scalability and interoperability of dynamic consent mechanisms on a broader scale.

Nevertheless, a notable aspect of this study was the simulation of real-world scenarios regarding the use of personalized options through the MyHealthHub application. Although the personalized option is an aspect of dynamic consent principles that safeguards individual autonomy in sharing and utilizing personal health data, it has received comparatively less attention than other features, such as withdrawal of consent, contactless communication, and unlimited communication. Participants in this study were not required to carefully read or view any particular page or information; rather, they used the application as usual and completed the assigned tasks by themselves. This statement underscores our endeavors to acquire a more realistic depiction of the circumstances in which individuals are anticipated to operate the application in the future. As evidenced by the fact that not all participants preferred to select personalized options each time rather than specifying conditions for automatic consent, it would appear that the intended reflection of a variety of realistic perspectives in this study was achieved, at least to some degree. Further research that juxtaposes the perspectives of active and passive individuals should provide a more holistic understanding of the effects of dynamic consent protocols on participation rates as well as whether such protocols yield more favorable outcomes while preserving individual autonomy.

## Conclusions

In a data-driven economy, personal health data facilitate progress in digital health ecosystems beyond their potential value as an asset. In digital health environments, dynamic consent is a promising strategy for protecting the autonomy and data sovereignty of individuals regarding their personal health data. The findings of this study indicate that by utilizing dynamic consent principles in the implementation of a digital consent application, individuals can be adequately informed regarding the manner in which their data are shared and used, thereby empowering them to make well-informed decisions. Participants highly valued the ability of digital interfaces to modify individual preferences in response to changing circumstances; this feature should be expanded to its fullest potential. Nevertheless, digital consent has certain challenges, such as apprehensions about the identification process and a lack of establishing trustworthy relationships with individuals. Therefore, while embracing the personalized and flexible advantages of the dynamic consent model, it is imperative to continuously contemplate technological and legal measures to ensure individual rights and privacy in the ever-evolving digital landscape.

### Supplementary Information

Additional file 1: Questionnaire

Additional file 2: Demographic characteristics of the study participants

Additional file 3: Validation results for the questionnaire items

## Data Availability

The datasets used and/or analysed during the current study are available from the corresponding author on reasonable request.

## Abbreviations

HIPAA: Health insurance portability and accountability act
GDPR: General data protection regulation
TAM: Technology Acceptance Model
SSO: Single sign-on
DUO: Data use ontology
GA4GH: Global alliance for genomics and health
FHIR: Fast healthcare interoperability resources
HL7: Health level seven
BPPC: Basic patient privacy consents
IHE: Integrating the healthcare enterprise
