# Supplementary material for: Opportunities and challenges of a dynamic consent-based application: personalized options for personal health data sharing and utilization
Source: BMC Med Ethics. 2024 Aug 31;25:92. doi: 10.1186/s12910-024-01091-3 (PMC11365279; doi:10.1186/s12910-024-01091-3)
Supplement: Supplementary file 1 — Additional file 1: Questionnaire [file 12910_2024_1091_MOESM1_ESM.pdf]

Additional File 1. Questionnaire (English version)

Part1. Participant demographics

Gender	○ Male	○ Female		
Age group	○ 18-30	○ 30s	○ 40s	○ Over 50
Education levels	○ High school<br>○ Associate degree<br>○ Bachelor's degree<br>○ Over master's degree			
Chronic disease	○ Yes	○ No		
Subjective health status	Very good **1 2 3 4 5** Very Bad			

Part2. Perceptions regarding the sharing and utilization of personal health data

1. Please indicate the extent to which you agree or disagree with the following statement: Sharing and utilizing personal health data is essential for the growth of the health care industry.

Strongly Disagree **1 2 3 4 5 6 7** Strongly Agree

2. When do you believe that consent is required to share your personal health data with other institutions?

○ Once (at the initial time of registering a specific service)
○ Partially (e.g., by providing automatic consent to pre-designated institutions)
○ For each time when sharing data

3. Please select all institutions with which you are willing to share personal health data for each data type.

	Family	Medical institutions	Research institutions	Public institutions	Private company	Insurance company
Basic	☐	☐	☐	☐	☐	☐
Physical	☐	☐	☐	☐	☐	☐
Mental	☐	☐	☐	☐	☐	☐
Medication	☐	☐	☐	☐	☐	☐
Genomics	☐	☐	☐	☐	☐	☐
Lifelogs	☐	☐	☐	☐	☐	☐

Part3. Acceptances of dynamic consent system

3a. Individual characteristics

Please indicate the extent to which you agree or disagree with the following statement.

3a-1. Health Literacy

HL-1. When visiting medical facilities, I am able to communicate with medical staff, such as physicians and nurses.

Strongly Disagree 1 2 3 4 5 6 7 Strongly Agree

HL-2. I am able to take medicines according to the instructions provided by physicians or pharmacists.

Strongly Disagree 1 2 3 4 5 6 7 Strongly Agree

HL-3. I am able to comprehend the dosage instructions printed on the medicine bag.

Strongly Disagree 1 2 3 4 5 6 7 Strongly Agree

3a-2. Health-related Interest

HI-1. My health is extremely essential to me.

Strongly Disagree 1 2 3 4 5 6 7 Strongly Agree

HI-2. I am interested in health-related issues and occasionally fine relevant articles or information.

Strongly Disagree 1 2 3 4 5 6 7 Strongly Agree

HI-3. Understanding one's own health status is essential for managing health.

Strongly Disagree 1 2 3 4 5 6 7 Strongly Agree

3b. System characteristics

3b-1. System usability

SU-1. I can readily use the MyHealthHub system's features, such as management, sharing, and utilization of personal health data.

Strongly Disagree 1 2 3 4 5 6 7 Strongly Agree

SU-2. I am capable of performing the specific tasks in the MyHealthHub system without assistance.

Strongly Disagree 1 2 3 4 5 6 7 Strongly Agree

SU-3. The MyHealthHub system provides uniform interfaces for comparable features.

Strongly Disagree 1 2 3 4 5 6 7 Strongly Agree

3b-2. System reliability

SR-1. I believe that my consent is required before using my personal health data through the MyHealthHub service and that it will not be used irresponsibly.

Strongly Disagree 1 2 3 4 5 6 7 Strongly Agree

SR-2. I believe it is feasible for MyHealthHub to protect the confidentiality of my personal health data.

Strongly Disagree 1 2 3 4 5 6 7 Strongly Agree

SR-3. I believe it is possible to protect the privacy of my personal information taking place within the MyHealthHub system.

Strongly Disagree **1 2 3 4 5 6 7** Strongly Agree

3c. TAM-derived variables

3c-1. Perceived usefulness

PU-1. MyHealthHub system will fulfill my requirements for sharing and utilizing my personal health data.

Strongly Disagree **1 2 3 4 5 6 7** Strongly Agree

PU-2. The MyHealthHub service will ensure my autonomy and control over the sharing and use of personal health data.

Strongly Disagree **1 2 3 4 5 6 7** Strongly Agree

PU-3. Compared to managing personal assets through banking services such as mobile internet banking, managing personal health data through the MyHealthHub system will be useful on a similar level.

Strongly Disagree **1 2 3 4 5 6 7** Strongly Agree

3c-2. Perceived ease of use

PE-1. The MyHealthHub system will be simple to use anywhere and at any time.

Strongly Disagree **1 2 3 4 5 6 7** Strongly Agree

PE-2. The MyHealthHub system will make it simple and convenient to manage personal health data.

Strongly Disagree **1 2 3 4 5 6 7** Strongly Agree

PE-3. Consenting (or not consenting) to the use of personal health data through the MyHealthHub system will be simple and completely functional.

Strongly Disagree **1 2 3 4 5 6 7** Strongly Agree

3c-3. Intention to use

IU-1. In comparison to managing personal assets via mobile or internet banking services, I believe the MyHealthHub system is essential at a comparable level for managing personal health data.

Strongly Disagree **1 2 3 4 5 6 7** Strongly Agree

IU-2. I am willing to use the MyHealthHub system to manage my personal health data.

Strongly Disagree **1 2 3 4 5 6 7** Strongly Agree

IU-3. I would recommend MyHealthHub system to my friends and family.

Strongly Disagree **1** **2** **3** **4** **5** **6** **7** Strongly Agree

Part4. Overall experiences

Please provide your opinions regarding the overall user experience evaluation, such as what needs to be improved regarding the MyHealthHub system or your opinions on the sharing and utilization of personal health data
