# Supplementary material for: Opportunities and challenges of a dynamic consent-based application: personalized options for personal health data sharing and utilization
Source: BMC Med Ethics. 2024 Aug 31;25:92. doi: 10.1186/s12910-024-01091-3 (PMC11365279; doi:10.1186/s12910-024-01091-3)
Supplement: Supplementary file 2 — Additional file 2: Demographic characteristics of the study participants [file 12910_2024_1091_MOESM2_ESM.pdf]

Additional File 2. Demographic characteristics of the study participants

Questionnaire	Responses
**Gender, n (%)**	
Men	22 (73.3%)
Women	8 (26.7%)
**Age group, n (%)**	
18-30	8 (26.7%)
31-40	15 (50.0%)
41-50	3 (10.0%)
Over 50s	4 (13.3%)
**Education levels, n (%)**	
High school	1 (3.3%)
Associate degree	0 (0.0%)
Bachelor's degree	19 (63.3%)
Over master's degree	10 (33.3%)
**Chronic disease, n (%)**	
Yes	2 (6.7%)
No	28 (93.3%)
**Subjective health status, n (%)**	
Very good	3 (10.0%)
Good	14 (46.7%)
Normal	12 (40.0%)
Bad	1 (3.3%)
Very bad	0 (0.0%)
