# Supplementary material for: Opportunities and challenges of a dynamic consent-based application: personalized options for personal health data sharing and utilization
Source: BMC Med Ethics. 2024 Aug 31;25:92. doi: 10.1186/s12910-024-01091-3 (PMC11365279; doi:10.1186/s12910-024-01091-3)
Supplement: Supplementary file 3 — Additional file 3: Validation results for the questionnaire items [file 12910_2024_1091_MOESM3_ESM.pdf]

Additional File 3. Validation results for the questionnaire items

The factor loading value is a correlation coefficient between a variable and a factor and ranges from -1 to 1. It is possible to determine whether each variable adequately explains the corresponding item. Each variable belongs to the factor that showed the highest correlation coefficient, and an absolute value over 0.4 indicates that the variable acceptably belongs to the factor. The factor loading values of variables used in this study ranged from 0.617 to 0.993, indicating that the items were well represented for each factor.[1]

Supplementary Table 1. Factor loading values of latent variables

	**Item**	**Components**						
		**1**	**2**	**3**	**4**	**5**	**6**	**7**
**Health literacy (HL)**	HL1	0.107	**0.812**	0.092	0.166	0.134	0.249	0.161
	HL2	0.410	**0.913**	0.185	0.061	0.308	0.290	0.103
	HL3	0.199	**0.907**	0.056	0.185	0.199	0.140	0.188
**Health interest (HI)**	HI1	**0.993**	0.268	-0.133	-0.320	-0.178	-0.015	-0.336
	HI2	**0.618**	0.224	-0.189	0.061	-0.105	0.127	-0.130
	HI3	**0.617**	0.317	0.036	-0.043	-0.036	0.102	-0.091
**System usability (SU)**	SU1	-0.260	0.070	0.511	0.701	0.649	0.381	**0.952**
	SU2	-0.383	0.217	0.489	0.614	0.632	0.206	**0.925**
	SU3	-0.273	0.190	0.527	0.691	0.665	0.423	**0.946**
**System reliability (SR)**	SR1	-0.004	0.155	0.644	0.673	0.827	**0.911**	0.416
	SR2	0.025	0.311	0.585	0.629	0.655	**0.971**	0.301
	SR3	-0.020	0.273	0.572	0.627	0.634	**0.971**	0.308
**Perceived usefulness (PU)**	PU1	-0.189	0.156	0.811	0.805	**0.929**	0.610	0.723
	PU2	-0.093	0.329	0.688	0.653	**0.862**	0.731	0.527
	PU3	-0.173	0.231	0.822	0.760	**0.959**	0.726	0.634
**Perceived ease of use (PE)**	PE1	-0.275	0.164	0.670	**0.825**	0.680	0.622	0.566
	PE2	-0.260	0.283	0.644	**0.915**	0.782	0.602	0.692
	PE3	-0.265	-0.071	0.610	**0.917**	0.683	0.577	0.631
**Intention to use (IU)**	IU1	-0.011	0.185	**0.930**	0.565	0.789	0.511	0.449
	IU2	-0.149	0.087	**0.971**	0.778	0.851	0.680	0.553
	IU3	-0.166	0.113	**0.980**	0.726	0.797	0.621	0.551

Supplemental Table 2 and Supplementary displays the results of internal consistency, concentration validity, and discriminant validity. The validity of questionnaire items for each group showed

acceptable results as shown in Supplementary Figure1. The Cronbach's alpha coefficient evaluates the internal consistency of questionnaire items for each group. It can range from 0 to 1, with values between 0.7 and 0.9 indicating an acceptable level of reliability and values greater than 0.9 indicating excellent reliability.[2] Three groups exhibited acceptable internal consistency: health literacy, health interest, and perceived ease of use, whereas the remaining four groups showed excellent internal consistency: system usability, system reliability, perceived usefulness, and intention to use.

Supplementary Table 2. The value of factor loading, Cronbach's alpha, construct reliability, and average variance extracted for each latent variable

	**Item**	**Factor loading**	**Cronbach's alpha**	**CR**	**AVE**
**Health literacy (HL)**	HL1	0.812	0.853	0.910	0.772
	HL2	0.913			
	HL3	0.907			
**Health interest (HI)**	HI1	0.993	0.783	0.799	0.583
	HI2	0.618			
	HI3	0.616			
**System usability (SU)**	SU1	0.952	0.935	0.959	0.885
	SU2	0.925			
	SU3	0.946			
**System reliability (SR)**	SR1	0.911	0.947	0.966	0.905
	SR2	0.971			
	SR3	0.971			
**Perceived usefulness (PU)**	PU1	0.929	0.906	0.941	0.842
	PU2	0.862			
	PU3	0.959			
**Perceived ease of use (PE)**	PE1	0.825	0.863	0.917	0.787
	PE2	0.915			
	PE3	0.917			
**Intention to use (IU)**	IU1	0.930	0.958	0.973	0.923
	IU2	0.971			
	IU3	0.980			

CR=Construct reliability; AVE=Average variance extracted

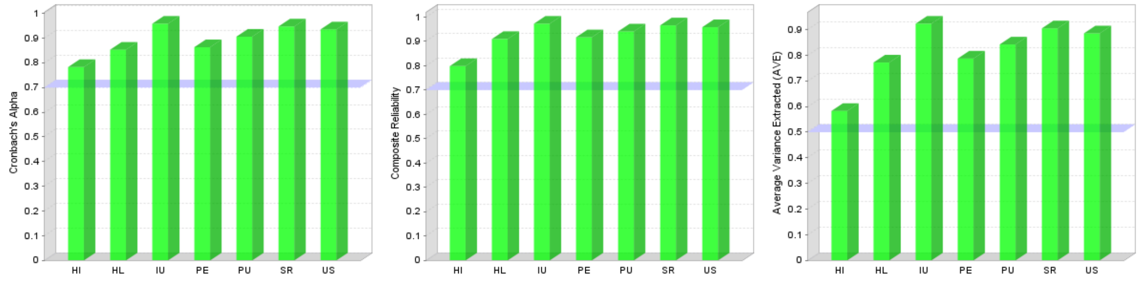

Supplementary Figure 1. Validation results for questionnaire items in each group: (a) internal consistency using Cronbach's alpha; (b) convergent validity using CR; and (c) discriminant validity using AVE value

Composite reliability (CR), also known as synthetic, construct or conceptual reliability, is a value used to estimate the reliability of convergent validity between questionnaire items for each group. Similar to Cronbach's alpha coefficient, its value can range from 0 to 1, with higher values indicating greater reliability. In general, a value greater than 0.7 can be interpreted as having acceptable validity.[3] All of the groups showed over 0.9 in the value of CR, except for one, health interest, with 0.799.

The value of the average variance extracted (AVE) was used to evaluate discriminant validity between groups. It can range from 0 to 1, and the higher the value, the greater the discriminant validity. All AVE values of groups defined in this study fell within the range of 0.583 to 0.923, confirming the discriminant validity of each group by displaying values greater than 0.5.[4] Discriminant validity can also be inferred when the correlation coefficient between each group is less than the square root of the AVE value.[5] According to Supplemental Table 3, all correlation coefficients were less than the AVE for each group.

Supplementary Table 3. The square root value of AVE and correlation coefficients between groups

	HL	HI	SU	SR	PU	PE	IU
**Health literacy (HL)**	**0.878**						
**Health interest (HI)**	0.291	**0.763**					
**System usability (SU)**	0.167	-0.322	**0.941**				
**System reliability (SR)**	0.257	0.000	0.361	**0.951**			
**Perceived usefulness (PU)**	0.254	-0.168	0.690	0.745	**0.918**		
**Perceived ease of use (PE)**	0.148	-0.301	0.712	0.678	0.809	**0.887**	
**Intention to use (IU)**	0.131	-0.117	0.541	0.633	0.846	0.724	**0.961**

References

1. Schaufeli, W.B., Bakker, A.B. and Salanova, M. The measurement of work engagement with a short questionnaire, *Educational and Psychological Measurement*, 2006; 66(4): 701–716. DOI:10.1177/0013164405282471.

2. Lance, C.E., Butts, M.M. and Michels, L.C. The sources of four commonly reported cutoff criteria, *Organizational Research Methods*, 2006; 9(2): 202–220. DOI:10.1177/1094428105284919.

3. Tentama, F., & Anindita, W. D. Employability scale: Construct validity and reliability. *International Journal of Scientific & Technology Research*, 2020; 9(4): 3166-3170.

4. Ahmad, S., Zulkurnain, N., & Khairushalimi, F. Assessing the validity and reliability of a measurement model in Structural Equation Modeling (SEM). *British Journal of Mathematics & Computer Science*, 2016; 15(3): 1-8.

5. Ab Hamid, M. R., Sami, W., & Sidek, M. M. Discriminant validity assessment: Use of Fornell & Larcker criterion versus HTMT criterion. *In Journal of Physics: Conference Series*, IOP Publishing, 2017; 890(1): p012163.
